# Supplementary material for: Nucleocapsid-specific T cell responses associate with control of SARS-CoV-2 in the upper airways before seroconversion
Source: Nat Commun. 2023 May 24;14:2952. doi: 10.1038/s41467-023-38020-8 (PMC10209201; doi:10.1038/s41467-023-38020-8)
Supplement: Supplementary file 1 — Supplementary Information [file 41467_2023_38020_MOESM1_ESM.pdf]

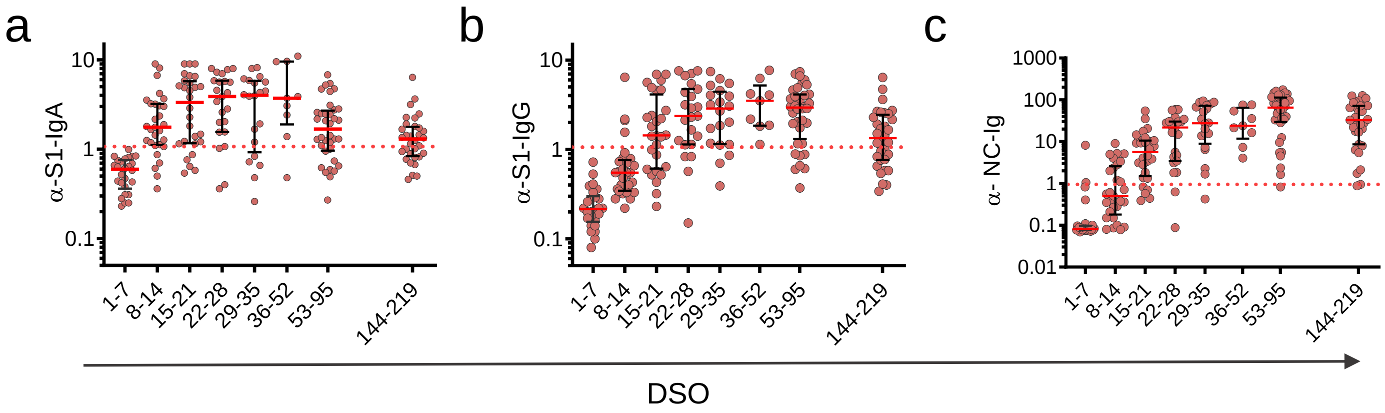

Supplementary Figure 1. Dynamics of antibody responses against SARS-CoV-2. (a-c) Antibody response dynamics are shown for S-specific IgA (ratio versus internal assay standard) (a), S-specific IgG (ratio versus internal assay standard) (b), and NC-specific total Ig in plasma (arbitrary units) (c). The cutoff for each assay is indicated by the dotted red line. Data are shown as median and IQR. DSO, days since symptom onset. Sample size at the different DSO: 1-7 DSO: n=25, 8-14 DSO: n= 30, 15-21 DSO: n= 28, 22-28 DSO: n=20, 29-35 DSO: n= 18, 36+ DSO: n=8, 60+ DSO: n=34, 180+ DSO: n=29.

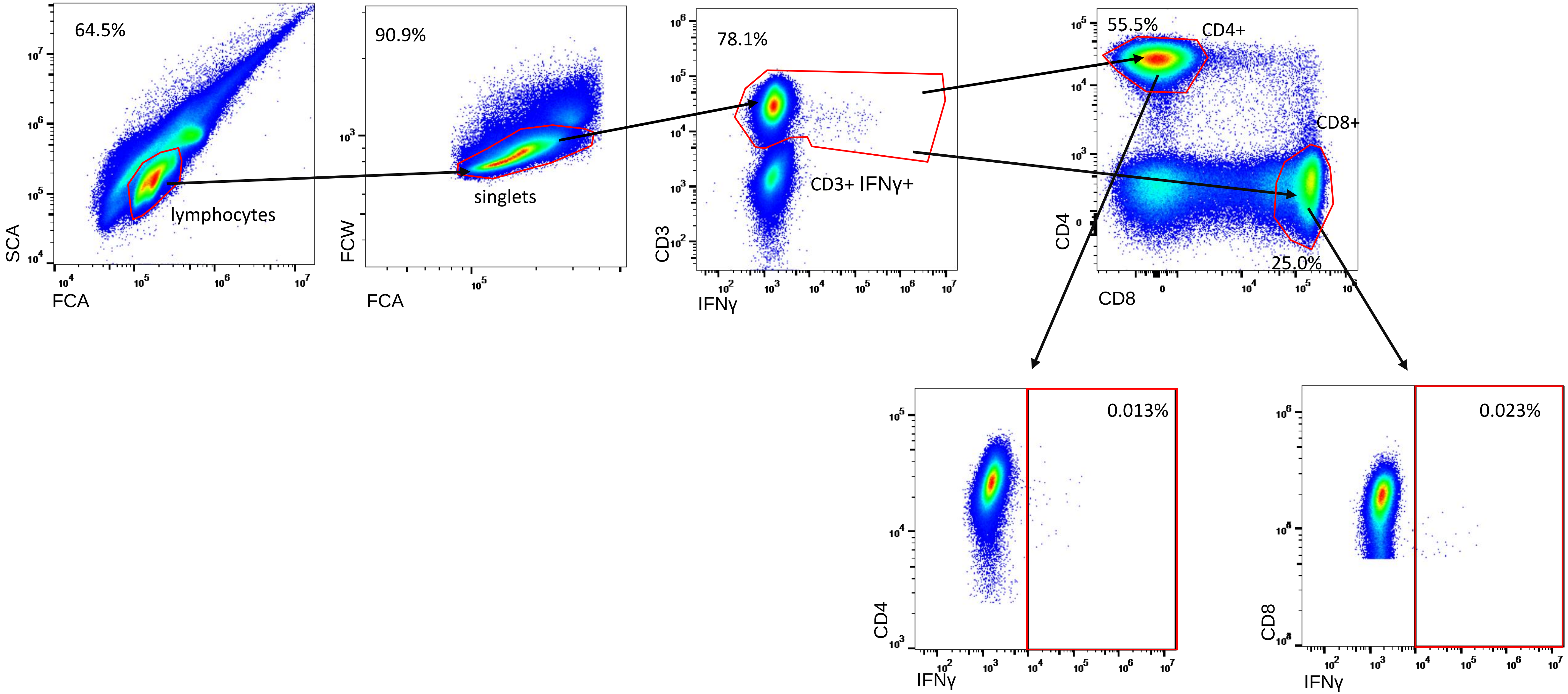

Supplementary figure 2: Gating strategy for gating NC or S- specific *IFNg* T cell

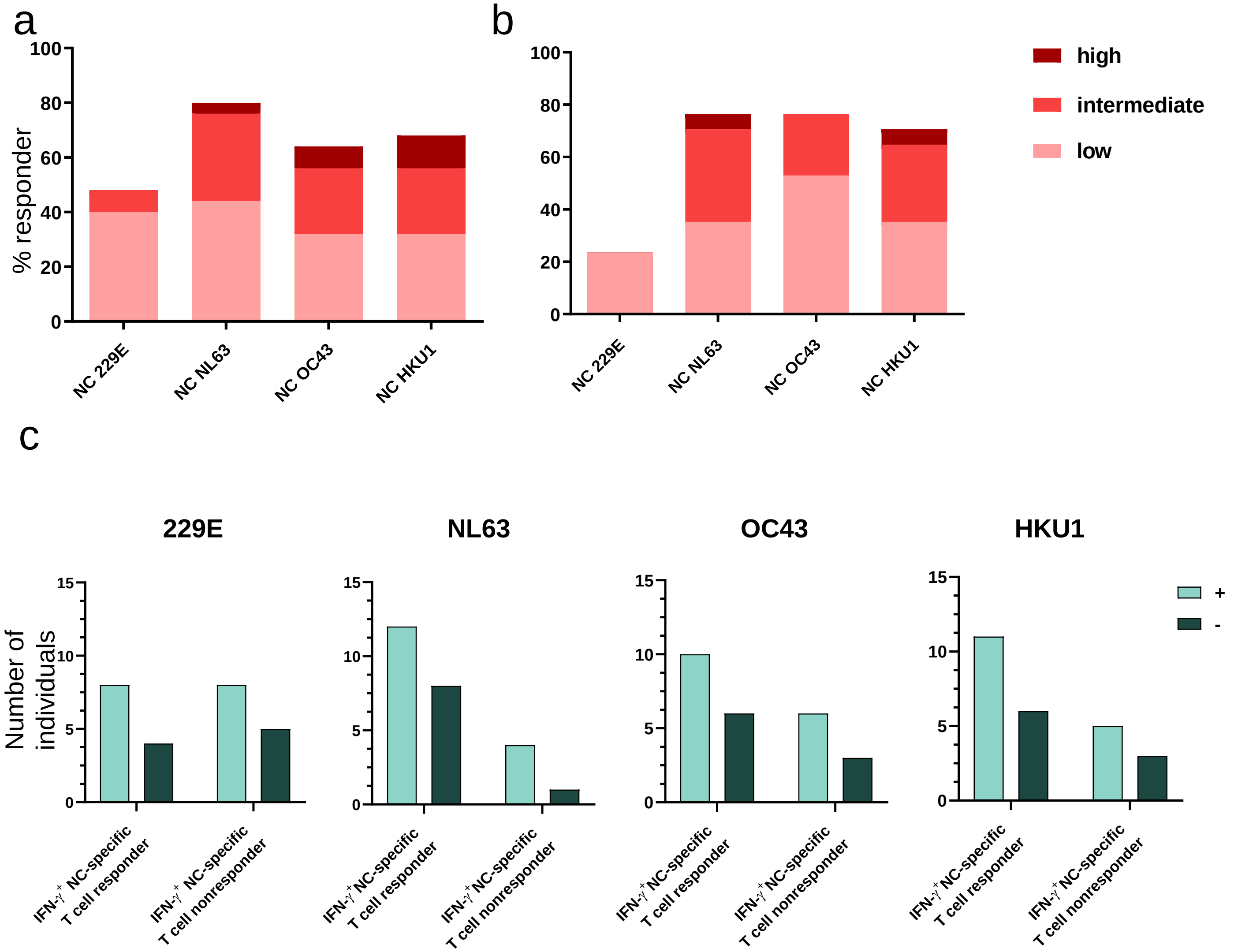

Supplementary Figure 3. Antibody responses against common cold coronaviruses. (a) Percent frequencies of responders (n = 24) with high (dark red), intermediate (red), and low NC-specific antibody responses (pink) against each of the four common cold coronaviruses (CCCVs) during the first week after symptom onset. (b) Percent frequencies of non-vaccinated healthy controls (n = 17) with high (dark red), intermediate (red), and low NC-specific antibody responses (pink) against each of the four CCCVs, as determined using manufacturer instructions. (c) Numbers of NC-specific T cell responders and non-responders with (light green) or without CCCV seroreactivity (dark green) during the first week after symptom onset. The number of patients for each condition was plotted and their distribution tested for significance using the Fishers exact test. None of the comparisons were significant ($p < 0.05$).

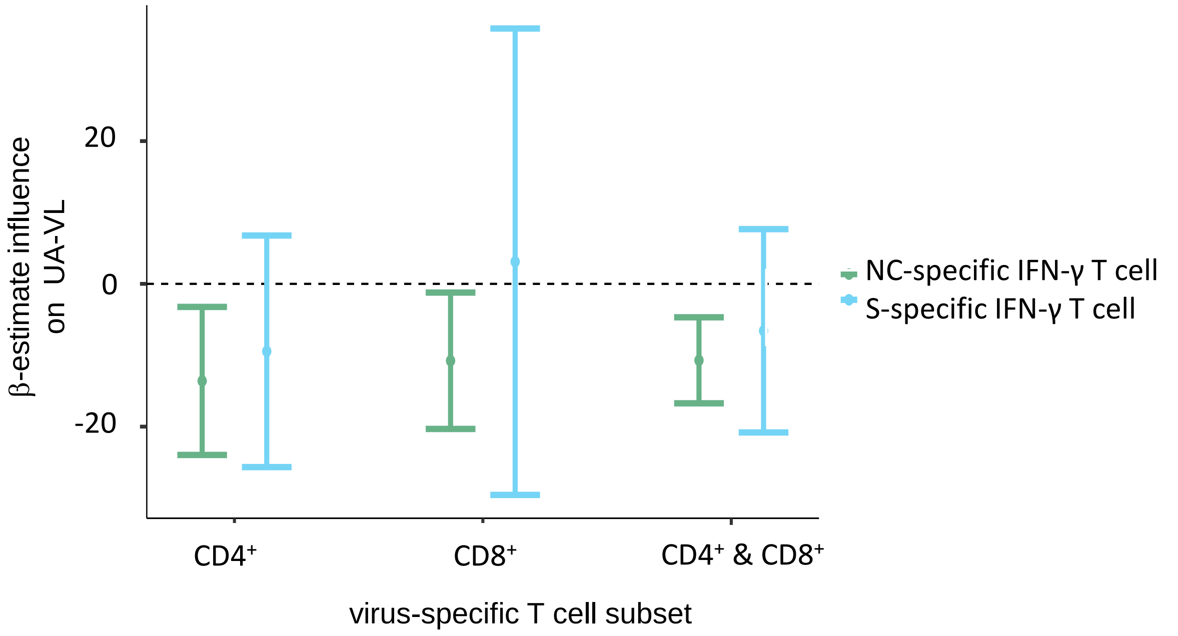

Supplementary Figure 4. Nucleocapsid-specific CD4$^+$ and CD8$^+$ T cell responses incrementally reduce the upper airways burden of SARS-CoV-2.

Summary of a censored linear mixed effects model showing the possible influence of SARS-CoV-2-specific T cells on upper airways viral loads (UA-Vls). Data from 33 individuals on different time points, yielding n=107 observations was studied. It was controlled for individual random effects and time fixed effects. Results are shown as β-estimates with 95% confidence intervals for NC-specific (green) and S-specific T cells (blue). Confidence intervals are computed using normal approximations, and are centered around the point estimates obtained from maximum likelihood estimation.

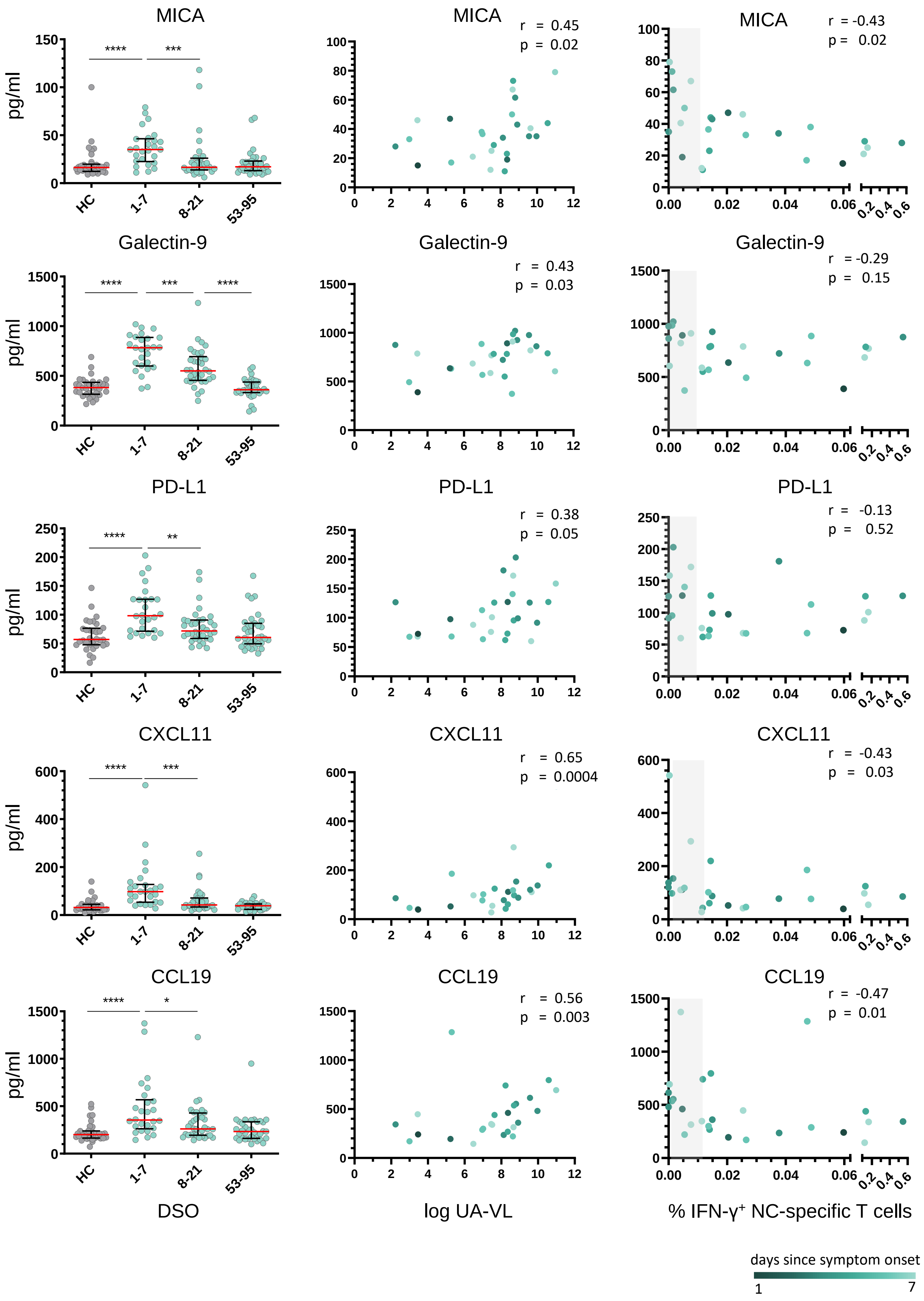

MICA
Galectin-9
PD-L1
CXCL11
CCL19
pg/ml
HC
1-7
8-21
53-95
DSO
r = 0.45
p = 0.02
r = -0.43
p = 0.02
r = 0.43
p = 0.03
r = -0.29
p = 0.15
r = 0.38
p = 0.05
r = -0.13
p = 0.52
r = 0.65
p = 0.0004
r = -0.43
p = 0.03
r = 0.56
p = 0.003
r = -0.47
p = 0.01
log UA-VL
% IFN-γ⁺ NC-specific T cells
days since symptom onset
1
7

Supplementary Figure 5. Dynamics of plasma soluble factors after infection with SARS-CoV-2. Left: plasma concentrations of MICA, Galectin-9, PD-L1, CXCL11, and CCL19 are shown for healthy controls (n = 17) and longitudinally for patients (0-7 DSO: n=25, 8-21 DSO: n=32, 60+ DSO: n=35). Data are shown as median and IQR. MICA ***p = 0.0003, ****p < 0.0001, Galectin-9 ***p = 0.0006, ****p < 0.0001, PD-1L **p = 0.0023, ****p < 0.0001; CXCL11 ***p = 0.001, ****p < 0.000; CCL19 *p < 0.02, ****p < 0.0001 (Mann-Whitney U test, two-sided test). Middle: Spearman rank correlations showing plasma soluble factors versus upper airways viral loads (UA-VLs) during the first week after symptom onset (key). Right: Spearman rank correlations showing plasma soluble factors versus nucleocapsid (NC)-specific T cell frequencies during the first week after symptom onset (key). The different shades of green in the middle and right panels indicate for each subject on which day during the first week after symptom onset the sample was collected. For spearman rank correlation the P-values (two-sided) is indicated in the graph

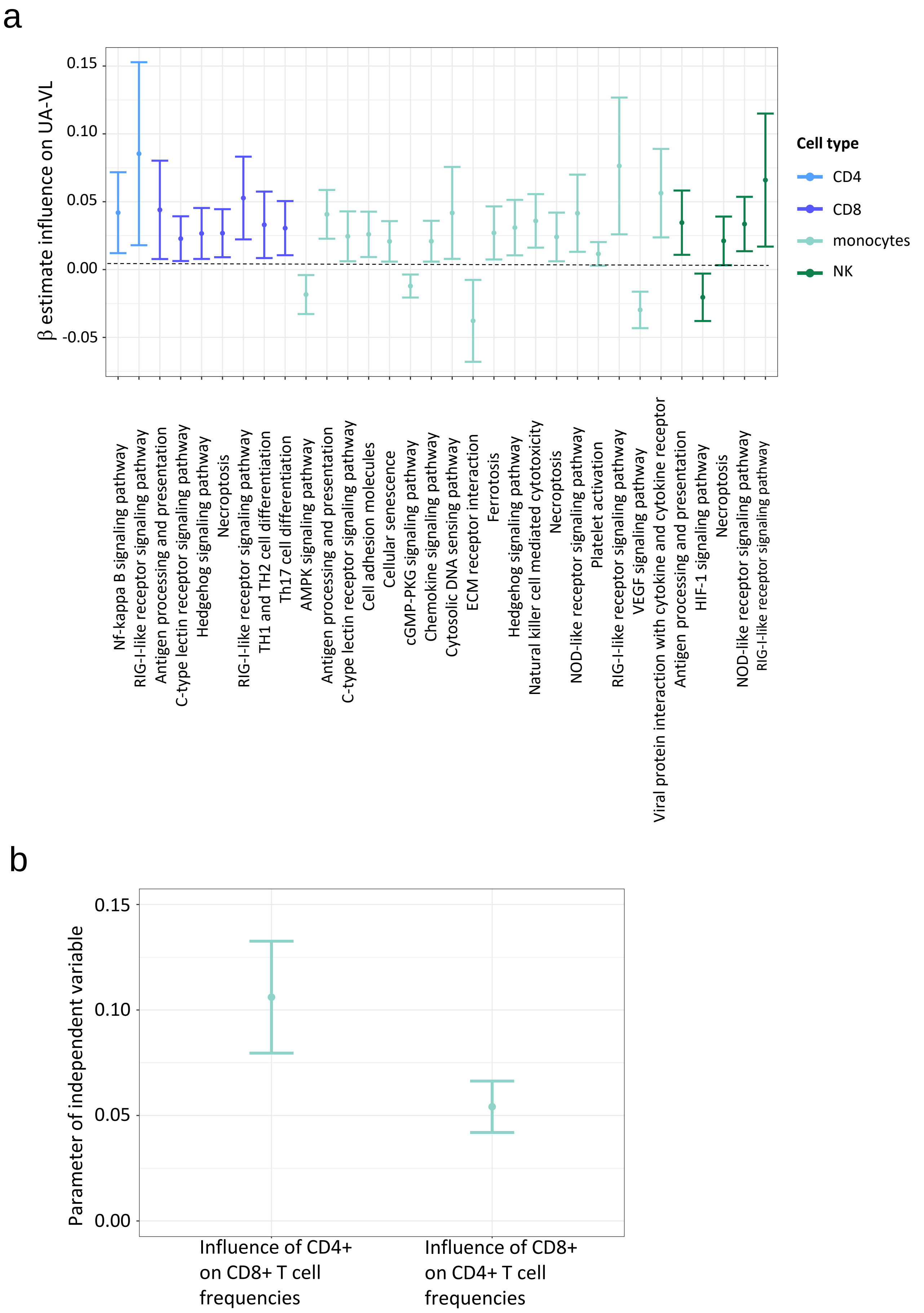
a
β estimate influence on UA-VL
0.15
0.10
0.05
0.00
-0.05
Cell type
CD4
CD8
monocytes
NK
Nf-kappa B signaling pathway
RIG-I-like receptor signaling pathway
Antigen processing and presentation
C-type lectin receptor signaling pathway
Hedgehog signaling pathway
Necroptosis
RIG-I-like receptor signaling pathway
TH1 and TH2 cell differentiation
Th17 cell differentiation
AMPK signaling pathway
Antigen processing and presentation
C-type lectin receptor signaling pathway
Cell adhesion molecules
Cellular senescence
cGMP-PKG signaling pathway
Chemokine signaling pathway
Cytosolic DNA sensing pathway
ECM receptor interaction
Ferrotosis
Hedgehog signaling pathway
Natural killer cell mediated cytotoxicity
Necroptosis
NOD-like receptor signaling pathway
Platelet activation
RIG-I-like receptor signaling pathway
VEGF signaling pathway
Viral protein interaction with cytokine and cytokine receptor
Antigen processing and presentation
HIF-1 signaling pathway
Necroptosis
NOD-like receptor signaling pathway
RIG-I-like receptor signaling pathway
b
Parameter of independent variable
0.15
0.10
0.05
0.00
Influence of CD4+ on CD8+ T cell frequencies
Influence of CD8+ on CD4+ T cell frequencies

Supplementary Figure 6. Modeling interactions between upper airways viral loads and activation of immune pathways in circulating immune cell subsets controlled for the influence of NC-specific T cell responses.

Data from 33 individuals on different time points, yielding n=107 observations was studied. Dots indicate point estimates, and whiskers indicate 95% confidence intervals. Confidence intervals are computed using normal approximations, and are centered around the point estimates obtained from maximum likelihood estimation. For hypothesis testing, we tested whether parameters are significantly different from zero using a two-sided t-test with 106 degrees of freedom and without multiple testing correction. (a) β-estimates for the interaction between upper airways viral loads (UA-VLs) and selected immune pathway scores achieving significance in the pre-selection ($p < 0.05$). Estimates were obtained using a censored linear mixed model. Results are clustered according to immune cell subsets (key). (b) Expected influence of NC-specific CD4+ T cells on NC-specific CD8+ T cell responses (left) and vice versa (right). Estimates were obtained using two separate linear mixed effect models and found to be significant ($p < 0.0001$). For detail p-values from supplementary figure 6a see supplementary table 5

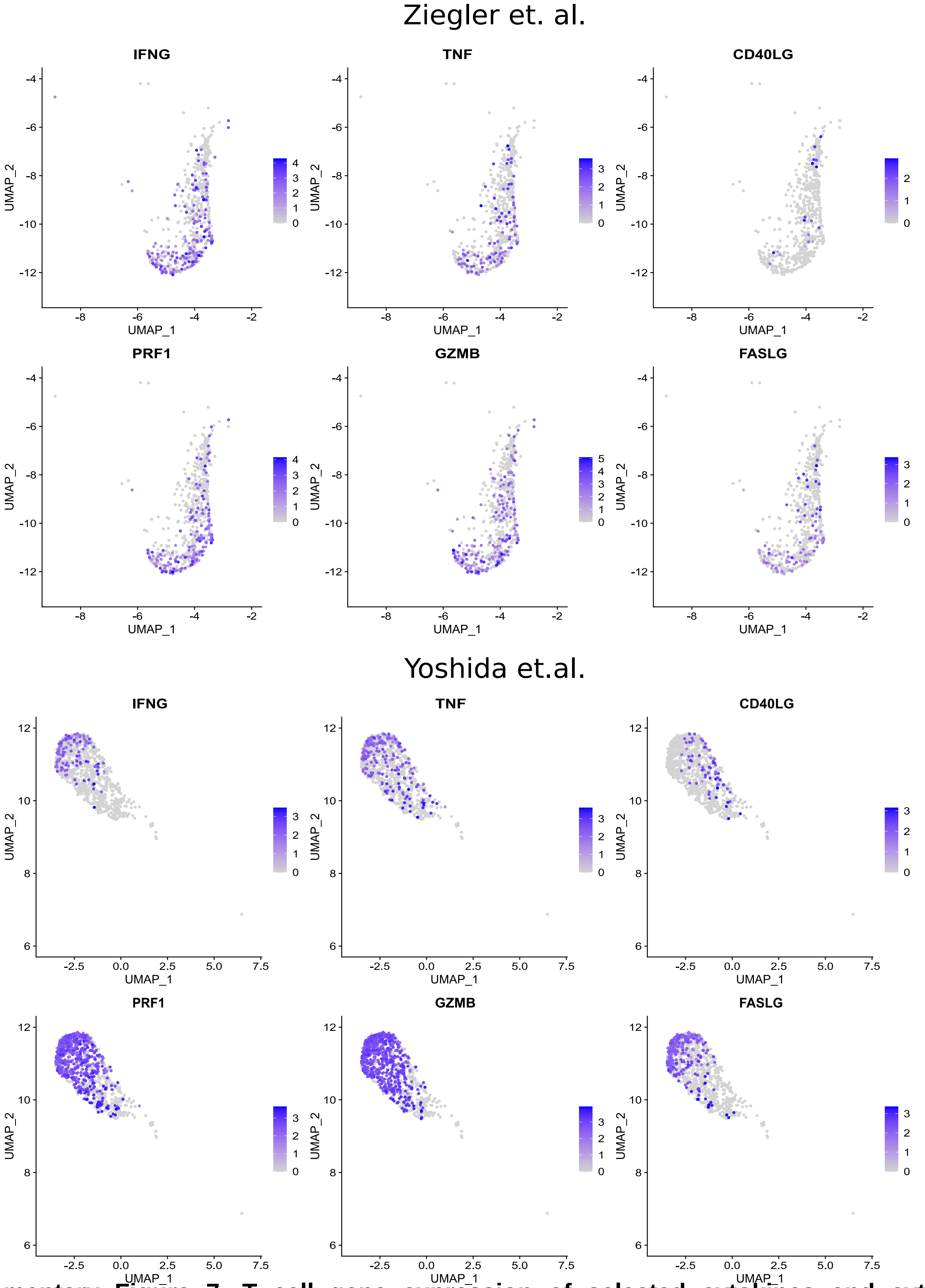

Supplementary Figure 7. T cell gene expression of selected cytokines and cytotoxic effector molecules in upper airway epithelia during acute SARS-CoV-2 infection. Expression of the indicated genes is shown for T cells from upper airway epithelia for two data sets (Ziegler and Yoshida). The color code indicates the number of mRNA transcripts detected for the indicated gene.

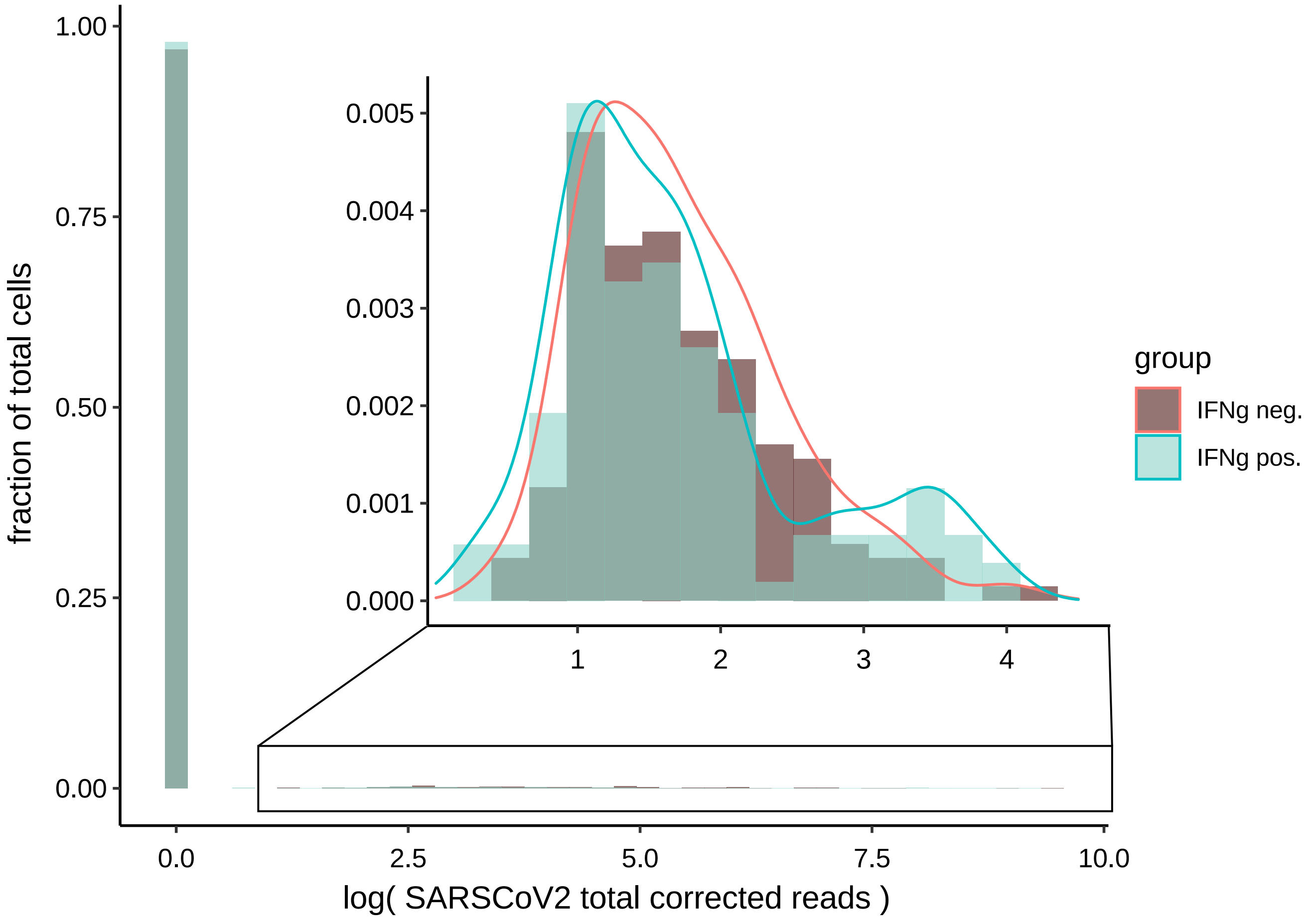

Supplementary Figure 8. Relationship between IFN-γ mRNA expression by T cells and detected SARS-CoV-2 RNA within nasopharyngeal epithelial cells. The graph shows the distribution of the logarithm of total corrected read counts of intracellular SARS-CoV-2 RNA in responders (blue) and non-responders (red). Responders with IFN-γ mRNA expressing T cells were characterized by lower counts and greater numbers of cells without SARS-CoV-2 RNA. The effect was significant ($p = 0.00013$).

Supplementary table 1. Exact p –values for figure 4 a

EIF2AK2

correlate	tissue	pvalue
vir	CD8	0.05
cd8ifn	CD8	0.422
cd4ifn	CD8	0.05
vir	CD4	0.0202
cd8ifn	CD4	0.3778
cd4ifn	CD4	0.0006
vir	NK	0.035
cd8ifn	NK	0.1054
cd4ifn	NK	0.0008
vir	Monocytes	0.1602
cd8ifn	Monocytes	0.5078
cd4ifn	Monocytes	0.015

OAS1

correlate	tissue	pvalue
vir	CD8	0.2398
cd8ifn	CD8	0.3567
cd4ifn	CD8	0.0351
vir	CD4	0.5729
cd8ifn	CD4	0.237
cd4ifn	CD4	0.1708
vir	NK	0.2334
cd8ifn	NK	0.4312
cd4ifn	NK	0.0053
vir	Monocytes	0.0544
cd8ifn	Monocytes	0.1133
cd4ifn	Monocytes	0.0021

STAT1

correlate	tissue	pvalue
vir	CD8	0.0202
cd8ifn	CD8	0.4784
cd4ifn	CD8	0.0159
vir	CD4	0.084
cd8ifn	CD4	0.8682
cd4ifn	CD4	0.0116
vir	NK	0.0064
cd8ifn	NK	0.1514
cd4ifn	NK	0.0244
vir	Monocytes	0.0522
cd8ifn	Monocytes	0.4979
cd4ifn	Monocytes	0.0058

Supplementary table 2. Exact p –values for figure 4 b

Description	p.value
NF-kappa B signaling pathway	0.0024
JAK-STAT signaling pathway	0.0033
Th17 cell differentiation	0.0053
B cell receptor signaling pathway	0.0109
Th1 and Th2 cell differentiation	0.0128
NOD-like receptor signaling pathway	0.0145
Cytokine-cytokine receptor interaction	0.0195
RIG-I-like receptor signaling pathway	0.0225
Fc gamma R-mediated phagocytosis	0.0258
PI3K-Akt signaling pathway	0.0279
T cell receptor signaling pathway	0.0286
TGF-beta signaling pathway	0.0342
Hematopoietic cell lineage	0.036
MAPK signaling pathway	0.0446
Ras signaling pathway	0.0456
Wnt signaling pathway	0.0467
Antigen processing and presentation	0.0523
Toll-like receptor signaling pathway	0.0558
Cell adhesion molecules	0.0676
ErbB signaling pathway	0.0734
Chemokine signaling pathway	0.0826
TNF signaling pathway	0.0826
Phospholipase D signaling pathway	0.0843
Hedgehog signaling pathway	0.0981
Natural killer cell mediated cytotoxicity	0.1036
Rap1 signaling pathway	0.1258
VEGF signaling pathway	0.1346
FoxO signaling pathway	0.1415
Leukocyte transendothelial migration	0.1821
Neutrophil extracellular trap formation	0.1933
Platelet activation	0.236
Fc epsilon RI signaling pathway	0.2392
ECM-receptor interaction	0.2425
Hippo signaling pathway	0.2804
Notch signaling pathway	0.2913
C-type lectin receptor signaling pathway	0.3062
IL-17 signaling pathway	0.3176
mTOR signaling pathway	0.3373
30.random	NA
300.random	NA

Supplementary table 3. Exact p –values for figure 4 c

Description	p.value
NF-kappa B signaling pathway	0.0202
Apelin signaling pathway	0.076
RIG-I-like receptor signaling pathway	0.2375
Platelet activation	0.3865
Ras signaling pathway	0.3821
TNF signaling pathway	0.3403
Neutrophil extracellular trap formation	0.3362
TGF-beta signaling pathway	0.3362
Cytosolic DNA-sensing pathway	0.3126
Natural killer cell mediated cytotoxicity	0.3049
Phospholipase D signaling pathway	0.2899
Neuroactive ligand-receptor interaction	0.2647
Cytokine-cytokine receptor interaction	0.2543
Leukocyte transendothelial migration	0.2342
Fc gamma R-mediated phagocytosis	0.231
Toll-like receptor signaling pathway	0.1914
ECM-receptor interaction	0.1914
Chemokine signaling pathway	0.1539
cAMP signaling pathway	0.1466
Viral protein interaction with cytokine and cytokine receptor	0.1174
Calcium signaling pathway	0.0943
Sphingolipid signaling pathway	0.053
Complement and coagulation cascades	0.0267
Fc epsilon RI signaling pathway	0.0233
VEGF signaling pathway	0.0083
30.random	NA
300.random	NA

Supplementary table 4. Exact p –values for figure 4 d

Description	p.value
cGMP-PKG signaling pathway	0.1755
30.random	NA
300.random	NA
Fc epsilon RI signaling pathway	0.3825
C-type lectin receptor signaling pathway	0.374
Antigen processing and presentation	0.3656
VEGF signaling pathway	0.3656
mTOR signaling pathway	0.3176
Natural killer cell mediated cytotoxicity	0.3099
Cell adhesion molecules	0.2877
Phospholipase D signaling pathway	0.2806
Cytokine-cytokine receptor interaction	0.2665
Th1 and Th2 cell differentiation	0.2463
Wnt signaling pathway	0.1973
IL-17 signaling pathway	0.1973
PI3K-Akt signaling pathway	0.1602
T cell receptor signaling pathway	0.1553
Chemokine signaling pathway	0.1553
Th17 cell differentiation	0.1323
ECM-receptor interaction	0.1281
Toll-like receptor signaling pathway	0.1198
TNF signaling pathway	0.1081
JAK-STAT signaling pathway	0.0544
Fc gamma R-mediated phagocytosis	0.0544
MAPK signaling pathway	0.0522
NOD-like receptor signaling pathway	0.05
B cell receptor signaling pathway	0.042
ErbB signaling pathway	0.035
RIG-I-like receptor signaling pathway	0.0237
NF-kappa B signaling pathway	0.0041

Supplementary table 5. Exact p –values for each coeffcent from supplementary figure 6a

pathway	name	p_value
CD4_hsa04622	RIG-I-like receptor signaling pathway	0.013061682
CD4_hsa04064	NF-kappa B signaling pathway	0.005843416
CD8_hsa04622	RIG-I-like receptor signaling pathway	0.000694107
CD8_hsa04625	C-type lectin receptor signaling pathway	0.006727923
CD8_hsa04612	Antigen processing and presentation	0.017345714
CD8_hsa04658	Th1 and Th2 cell differentiation	0.005486681
CD8_hsa04659	Th17 cell differentiation	0.00271064
CD8_hsa04340	Hedgehog signaling pathway	0.005486681
CD8_hsa04217	Necroptosis	0.002999916
Mono_hsa04611	Platelet activation	0.009198027
Mono_hsa04621	NOD-like receptor signaling pathway	0.00421168
Mono_hsa04622	RIG-I-like receptor signaling pathway	0.002960069
Mono_hsa04623	Cytosolic DNA-sensing pathway	0.015607369
Mono_hsa04625	C-type lectin receptor signaling pathway	0.008933603
Mono_hsa04650	Natural killer cell mediated cytotoxicity	0.000361973
Mono_hsa04612	Antigen processing and presentation	8.99882E-06
Mono_hsa04062	Chemokine signaling pathway	0.006448565
Mono_hsa04061	Viral protein interaction with cytokine and cytokine receptor	0.000710879
Mono_hsa04512	ECM-receptor interaction	0.014123945
Mono_hsa04514	Cell adhesion molecules	0.002356341
Mono_hsa04340	Hedgehog signaling pathway	0.003002655
Mono_hsa04370	VEGF signaling pathway	1.44629E-05
Mono_hsa04022	cGMP-PKG signaling pathway	0.005045123
Mono_hsa04152	AMPK signaling pathway	0.012071256
Mono_hsa04216	Ferroptosis	0.006773564
Mono_hsa04217	Necroptosis	0.008614264
Mono_hsa04218	Cellular senescence	0.006292951
NK_hsa04621	NOD-like receptor signaling pathway	0.001026402
NK_hsa04622	RIG-I-like receptor signaling pathway	0.008359055
NK_hsa04612	Antigen processing and presentation	0.004235453
NK_hsa04066	HIF-1 signaling pathway	0.021909713
NK_hsa04217	Necroptosis	0.020659667

CD4: CD4 Tcell; CD8: CD8 Tcell; Mono: Monocyten; NK: Natural killer cells
