# Supplementary material for: Nucleocapsid-specific T cell responses associate with control of SARS-CoV-2 in the upper airways before seroconversion
Source: Nat Commun. 2023 May 24;14:2952. doi: 10.1038/s41467-023-38020-8 (PMC10209201; doi:10.1038/s41467-023-38020-8)
Supplement: Supplementary file 3 — Additional Supplementary Information [file 41467_2023_38020_MOESM3_ESM.pdf]

<u>Description of additional supplementary files</u>

Supplementary Dataset 1 Ziegler_DifferentialGeneExpression_allCellTypes.xlsx Differentially expressed genes within all nasopharyngeal epithelial cell type clusters between acutely SARS-CoV-2 infected subjects with and without T cells expressing IFN-γ mRNA from the Ziegler dataset.

Supplementary Dataset 2 Ziegler_pathwayEnrichment_allCellTypes.xlsx Enrichment of a subset of KEGG pathway of all nasopharyngeal epithelial cell type clusters between acutely SARS-CoV-2 infected subjects with and without T cells expressing IFN-γ mRNA from the Ziegler dataset. The pathways were limited to those contained in the BRITE categories Signal Transduction, Signaling Molecules and Interaction, Immune System, and Cell Growth and Death.

Supplementary Dataset 3 Ziegler_goEnrichment_allCellTypes.xlsx Enrichment of a Gene Onthology terms of all nasopharyngeal epithelial cell type clusters between acutely SARS-CoV-2 infected subjects with and without T cells expressing IFN-γ mRNA from the Ziegler dataset.

Supplementary Dataset 4 Yoshida_DifferentialGeneExpression_allCellTypes.xlsx Differentially expressed genes within all nasal epithelial cell type clusters between acutely SARSCoV-2 infected subjects with and without T cells expressing IFN-γ mRNA from the Yoshida dataset.

Supplementary Dataset 5 Yoshida_pathwayEnrichment_allCellTypes.xlsx Enrichment of a subset of KEGG pathway of all nasal epithelial cell type clusters between acutely SARS-CoV-2 infected subjects with and without T cells expressing IFN-γ mRNA from the Yoshida dataset. The pathways were limited to those contained in the BRITE categories Signal Transduction, Signaling Molecules and Interaction, Immune System, and Cell Growth and Death.

Supplementary Dataset 6 Yoshida_goEnrichment_allCellTypes.xlsx Enrichment of a Gene Onthology terms of all nasal epithelial cell type clusters between acutely SARS-CoV-2 infected subjects with and without T cells expressing IFN-γ mRNA from the Yoshida dataset.

Supplementary Dataset 7 GenesOfInterest_tcells.xlsx mRNA expression of selected set of cytokines, effector molecules and other genes of interest in T cells of the upper airway epithelia. The table shows percentage of T cells and absolute numbers of acutely SARS-CoV-2 infected subjects which express respective genes in Ziegler and Yoshida data sets.
